# Development and Molecular Cytogenetic Characterization of a Novel Wheat-Rye T6RS.6AL Translocation Line from *Secale cereale* L. Qinling with Resistance to Stripe Rust and Powdery Mildew

**DOI:** 10.3390/ijms231810495

**Published:** 2022-09-10

**Authors:** Tianheng Ren, Zixin Sun, Zhenglong Ren, Feiquan Tan, Peigao Luo, Zhi Li

**Affiliations:** 1 State key Laboratory of Crop Gene Exploration and Utilization in Southwest China, Sichuan Agricultural University, Wenjiang, Chengdu 611130, China; 2 College of Agronomy, Sichuan Agricultural University, Wenjiang, Chengdu 611130, China; 3 Provincial Key Laboratory for Plant Genetics and Breeding, Wenjiang, Chengdu 611130, China

**Keywords:** wheat, chromosome translocation, 6RS.6AL, Qinling rye, ND-FISH, disease resistance

## Abstract

In this study, a novel T6RS.6AL translocation line, 117-6, was selected from a cross between common Chuannong25 (CN25) wheat and Qinling rye. The results of nondenaturing fluorescence in situ hybridization (ND-FISH) and PCR showed that 117-6 contained two T6RS.6AL translocation chromosomes. The distal region of the 6RS chromosome in 117-6 was mutant and showed different FISH signal patterns. When inoculated with different stripe rust races and powdery mildew races in seedlings, 117-6 expressed high resistance to them. The 117-6 line also exhibited high resistance to stripe rust and powdery mildew in the field under natural *Puccinia striiformis* f. sp. *tritici* (*Pst*) and *Blumeria graminis* f. sp. *tritici* (*Bgt*) infection. The cytogenetic analysis indicated that the introduction of 6RS conferred resistance ability. Compared with wheat parent CN25, 117-6 exhibited excellent agronomic traits in the field. The present study indicated that Qinling rye may carry favorite genes as a potential source for wheat genetic improvement, and 117-6 could be a useful germplasm for wheat breeding programs in the future.

## 1. Introduction

Common wheat (*Triticum aestivum* L.) is one of the most important crops in the world and provides approximately 20% of the food in the human diet [1]. It is very important to maintain and continue increasing the yield of common wheat to relieve the pressure exerted by the decrease in farmland and the increase in the population [2]. However, stripe rust and powdery mildew, caused by *Puccinia striiformis* Westend. f. sp. *tritici* ERikss. (*Pst*) and *Blumeria graminis* f. sp. *tritici* (*Bgt*), respectively, are considered to be devastating wheat diseases worldwide, and are very detrimental to wheat production in subtropical agricultural zones [3,4]. In some of the most prevalent areas, stripe rust and powdery mildew can result in the loss of approximately 20–30% of wheat production [4,5]. China is the country with the highest wheat production in the world [5]. Stripe rust and powdery mildew are the most severe diseases, and there are epidemics almost every year in China, seriously affecting wheat production. For example, in 1950, 1964, 1990, and 2002, there were four violent epidemics of *Pst*, causing losses of 6.0, 3.2, 1.8, and 1.3 million tons of yield, respectively [6]. In 1990 and 1991, more than 12 million hectares of wheat were attacked by *Bgt*, causing 1.4 million and 0.7 million tons of yield losses, respectively [7].

Distant hybridization (wide crosses) is an effective and rapid way to improve the genetic basis of wheat [8]. There are a large number of useful genes in wheat-related species, and alien chromosomes carrying these genes exist in the wheat genome in the form of addition, substitution, and translocation through distant hybridization [8,9,10]. Therefore, many chromosome fragments of wheat-related species, such as rye (*Secale cereale*) [9,10], *Haynaldia villosa* [11], *Elytrigia intermedium* [12], *Agropyron cristatum* [13,14], *Psathyrostachys huashanica* [15], *Aegilops* [16], *Thinopyrum intermedium* [17], *Leymus mollis* [18], etc. were introduced into the wheat genome through distant hybridization in the last few decades. However, the most important and valuable related species for the improvement of wheat genetics is rye [4,8]. The resistance genes *Yr9* (for stripe rust), *Pm8* (for powdery mildew), *Lr26* (for leaf rust), and *Sr31* (for stem rust), as well as yield-enhanced genetic and environmental adaptability factors, were introduced into the wheat genome through the T1RS.1BL translocation chromosome [19,20,21,22]. Therefore, T1RS.1BL translocations have been used widely in wheat breeding programs worldwide, and thousands of wheat cultivars containing T1RS.1BL translocation chromosomes were released [23,24]. However, due to the new prevalence of virulent pathogens, the resistance genes located on the 1RS chromosome, which came from the German Petkus rye, were overcome [4,5,8]. Rye is a cross-pollinated plant with high genetic diversity within the population of a single rye variety [25]. Therefore, many new sources of 1RS chromosomes have been introduced into the wheat genome, and have been found to exhibit high genetic diversity for disease resistance [26,27]. In addition to the 1RS chromosome of rye, other chromosomes of rye also contain a large number of favorable genes for wheat genetic improvement. For example, 1909 disease resistance-associated genes were mapped to the seven assembled chromosomes of Weining rye, only 242 of which were found on the 1R chromosome [28]. Therefore, many researchers are focusing their attention on chromosomes 2R to 7R of rye. For example, Liu et al. [29] reported that a 3RL addition line exhibited high resistance to stem rust (Ug99). Ren et al. [30] reported that a 7RL.7BS translocation line exhibited resistance to stripe rust, powdery mildew, and *Fusarium* head blight. However, the carrier chromosomes of most alien resistance genes are often linked with poor agronomic or quality traits. In addition to 1RS, only a few alien chromosomes or genes have been used in wheat breeding programs. The 6R chromosome of rye contains many useful genes for wheat genetic improvement, such as resistance genes for pests (*H25*) [31], powdery mildew (*Pm20* and *Pm56*) [32,33], stripe rust (*Yr83*) [34,35], and dwarfing [36]. However, most of the favorite genes of 6R were located on the long chromosome arm (6RL), and most of the 6R chromosomes existed in the wheat genome in the form of chromosome additions [9,37]. Moreover, fewer studies have been conducted on 6RS than on 6RL, and there are also no reports of 6RS resistance to both stripe rust and powdery mildew.

In this study, a new 6RS.6AL wheat-rye translocation line, 117-6, was developed and selected from crosses between Chuannong25 (CN25) wheat and Qinling rye. The 117-6 line exhibited high resistance to stripe rust and powdery mildew, and it also showed great agronomic traits, making it a valuable genetic resource for wheat breeding programs.

## 2. Results

### 2.1. Development and Identification of the New T6RS.6AL Translocation Line 117-6

**Figure 1:**
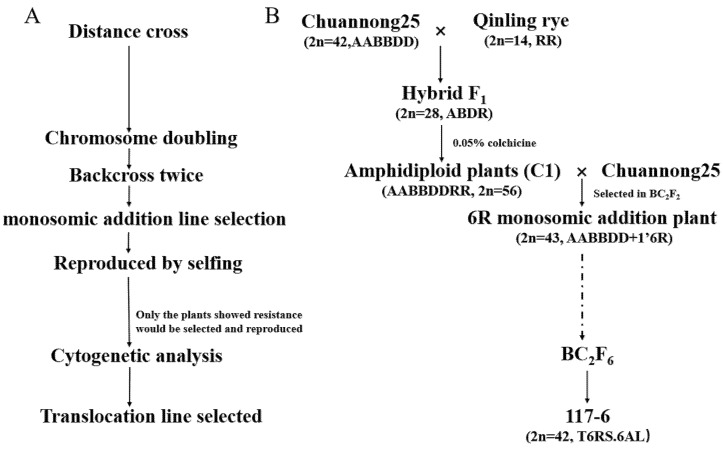
Development of new T6RS.6AL translocations line 117-6. (**A**) The development of the translocation 117-6. (**B**) Pedigree of 117-6.

The pedigree of the T6RS.6AL translocation line 117-6 is displayed in Figure 1.

**Figure 2:**
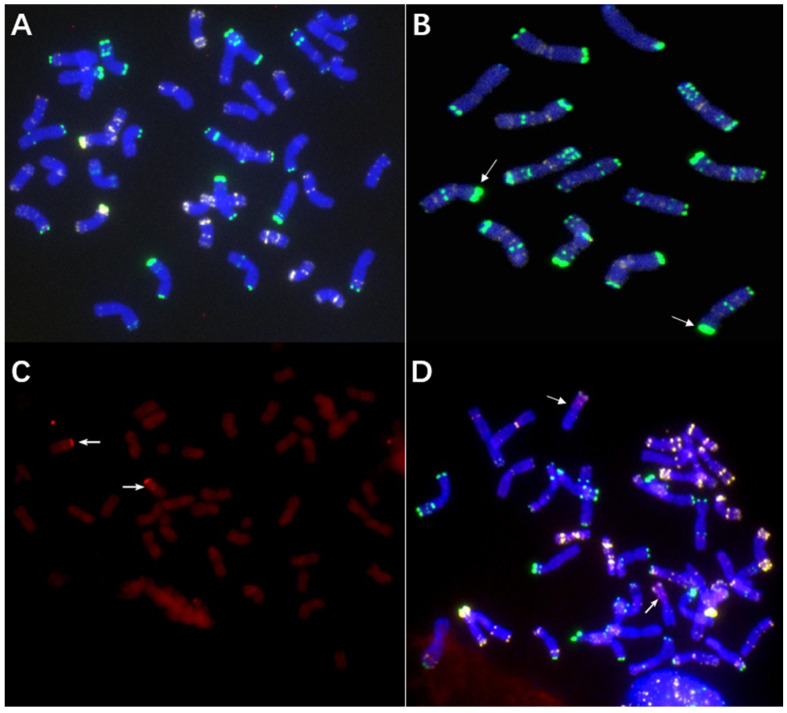
Cytogenetic identification (600×) of the T6RS.6AL translocation line 117-6. (**A**) Chromosome structure of CN25. No rye chromatin was found. (**B**) Chromosome structure of Qinling rye. The arrows show the strong signal patterns of the distal region of 6RS. (**C**) GISH results of 117-6; the arrows show the translocation chromosomes. (**D**) ND-FISH results of 117-6. The arrows show the T6RS.6AL translocation chromosomes. Oligo-pSc119.2-1: green, Oligo-pTa535-1: white, Oligo-Ku, Oligo-pSc200, and Oligo-pSc250: red. The wheat chromosomes were blue in (**A**,**B**,**D**), and dark red in (**C**). The rye chromosomes were bright red in C.

The wheat parent CN25 (2n = 42, AABBDD) (Figure 2A) was directly crossed with Qinling rye (2n = 14, RR) (Figure 2B), and several F_0_ seeds (2n = 28, ABDR) were obtained. Then, the growing points of seedlings of the F_1_ plants were treated with colchicine plus dimethyl sulfoxide to produce amphidiploids (C_1_, 2n = 56, AABBDDRR). The C_1_ plants showed high resistance to stripe rust and powdery mildew in the field under natural infection. The BC_2_F_2_ generation was obtained using backcross, and a 6R monosomic addition plant (2n = 43, AABBDD + 1′6R) was selected via nondenaturing fluorescence in situ hybridization (ND-FISH). This plant showed high resistance to stripe rust and powdery mildew in the field. In the BC_2_F_6_ generation, 40 seeds were randomly selected and cultured in the culture room of the laboratory. The root tips of the seedlings were used for cytogenetic analysis. Then, the surviving seedlings were transplanted to the field. In these examined plants, one plant containing a pair of T6RS.6AL translocation chromosomes was selected and named 117-6. Genomic fluorescence in situ hybridization (GISH) showed that 117-6 contained a pair of wheat-rye translocation chromosomes (Figure 2C). The combination of the probes Oligo-pSc119.2-1, Oligo-pTa535-1, Oligo-Ku, Oligo-pSc200, and Oligo-pSc250 indicated that 117-6 contained a pair of T6RS.6AL translocation chromosomes (Figure 2D). Usually, the signal patterns of pSc119.2 on the distal region of 6RS are very strong (Figure 2B). However, in this study, the signal patterns of pSc119.2 of the T6RS.6AL translocation line 117-6 were much weaker than those shown in the rye genome (Figure 2B,D). It was suggested that the chromosome structure of the distal region of the 6RS arm in 117-6 had variation.

**Figure 3:**
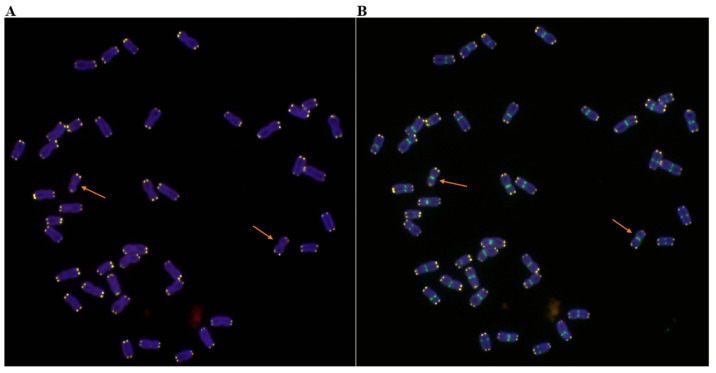
Cytogenetic identification (600×) of the centromere and telomere of T6RS.6AL translocation line 117-6. (**A**) The signals of Oligo-PAWRC.1 (red), and Oligo-Telo (white). (**B**) The signals of Oligo-CCS1 (green), and Oligo-Telo (white). The signal patterns were derived from one cell. The wheat chromosomes were blue. The arrows showed the T6RS.6AL translocation chromosomes.

The centromere and telomere composition of the 117-6 was identified using the combinations of the probes Oligo-CCS1, Oligo-PAWRC.1, and Oligo-Telo. The centromere of 117-6 showed both signals of Oligo-CCS1 and Oligo-PAWRC.1 at the same place (Figure 3). Additionally, all chromosomes of 117-6 showed complex signals of Oligo-Telo (Figure 3). The results indicated that 117-6 is a full arm 6RS.6AL translocation line.

**Figure 4:**
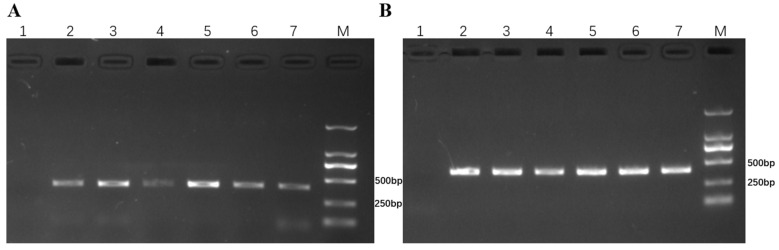
PCR results from primers KU88 and KU291. (**A**) PCR results from primer KU88. (**B**) PCR results from primer KU291. Lane 1: CN25 (wheat parent); Lane 2: Qinling rye; Lanes 3 and 4: 117-6 (T6RS.6AL translocation); 5: 6RS addition plant; 6: 6R monosomic addition plant; 7: heterozygous. M: marker DL2000.

PCR was also used to identify the 6RS chromosome of the new translocation line 117-6. The results of the PCR analysis showed that the new T6RS.6AL translocation line 117-6 could amplify the expected band at approximately 400 bp, while the wheat parent CN25 could not amplify the expected fragment band (Figure 4).

117-6 exhibited a high resistance to stripe rust and powdery mildew in the field, and exhibited some excellent agronomic traits, such as more spikes. The seeds of this plant were harvested and then sown in the next crop season. The progeny showed consistent morphological characteristics. The cells of the root tips of the continued generation were also examined using ND-FISH (10 randomly selected plants per generation, a total of three generations were examined), which indicated that the T6RS.6AL translocation chromosomes could be inherited normally. These results indicated that 117-6 was a stable homozygous T6RS.6AL translocation line.

### 2.2. Analysis of Resistance to Stripe Rust and Powdery Mildew

**Figure 5:**
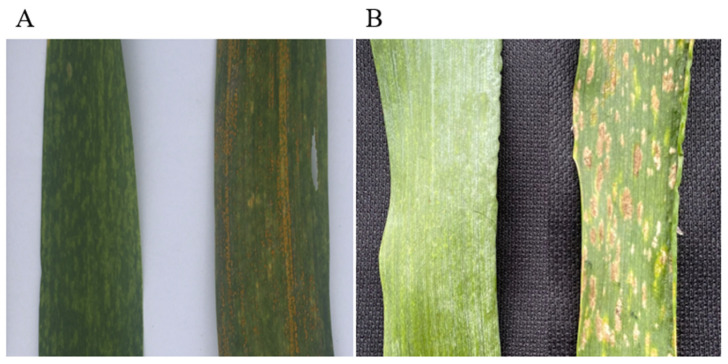
Resistance of translocation line 117-6 to stripe rust and powdery mildew in the field. (**A**) Resistance to stripe rust; (**B**) Resistance to powdery mildew. Left: 117-6, right: CN25.

The wheat parent CN25 was intermediate resistant to two *Pst* races (CYR32 and 34), highly susceptible to the *Bgt* E20 race, exhibited intermediate resistance to stripe rust, and was highly susceptible to powdery mildew in the field (Table 1). On the other hand, Qinling rye was highly resistant to three *Pst* races and to the *Bgt* E20 race. Qinling rye also exhibited high resistance to stripe rust and powdery mildew in the field (Table 1). The seedlings of the new T6RS.6AL translocation line 117-6 showed high resistance to three *Pst* races and to the *Bgt* E20 race. In addition, 117-6 also showed high resistance to stripe rust and powdery mildew in the field during the adult stage (Figure 5).

**Figure 6:**
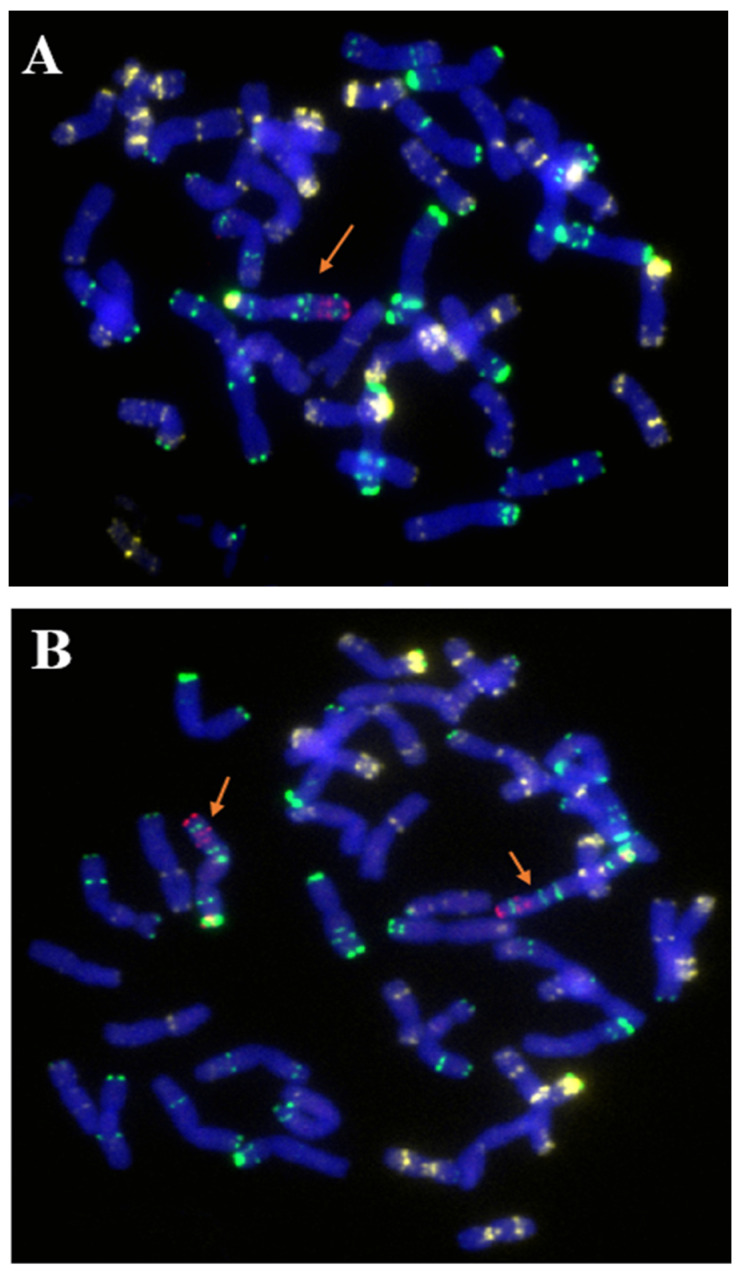

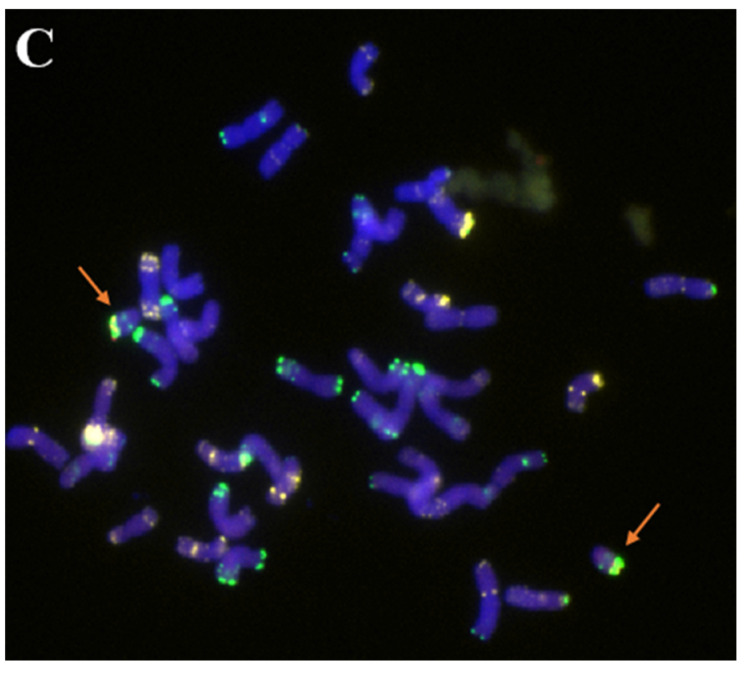
Cytogenetic identification (600×) in the BC_2_F_6_ generation. (**A**) 6R monosomic addition plant; the arrow shows the 6R chromosome. (**B**) 6R disomic addition plant; the arrows show the 6R chromosomes. (**C**) 6RS addition plant; the arrows show the 6RS chromosome arms. Oligo-pSc119.2-1: green, Oligo-pTa535-1: white, Oligo-Ku, Oligo-pSc200, and Oligo-pSc250: red. The chromosomes were blue.

In addition to the homozygous T6RS.6AL translocation line 117-6, many other types of plants with 6R or 6RS were also obtained (Figure 4 and Figure 6), such as heterozygous T6RS.6AL translocation plants (2n = 42, 1′6A + 1′T6RS.6AL), 6R monosomic addition plants (2n = 43, AABBDD + 1′6R) (Figure 4 and Figure 6A), the 6R disomic addition line (2n = 44, AABBDD + 1″6R) (Figure 4 and Figure 6B), 6RS addition plants (2n = 43, AABBDD+ 1′6RS or 2n = 44, AABBDD + 1″6RS) (Figure 4 and Figure 6C), and the plants that lost 6R (2n = 42, AABBDD), which were also identified in the BC_2_F_6_ generation. However, 117-6, 6RS.6AL heterozygous translocation lines, and 6R or 6RS addition lines exhibited resistance to stripe rust and powdery mildew in the field. On the other hand, the plants that lost their 6R chromosome also lost their resistance to stripe rust and powdery mildew (Table 2). These results indicated that the 6RS chromosome arm provided resistance to stripe rust and powdery mildew.

### 2.3. Agronomic Traits of the T6RS.6AL Translocation Line 117-6

Compared with the wheat parent CN25, the T6RS.6AL translocation line 117-6 showed significantly enhanced agronomic traits (Table 3). In the 2020 to 2021 crop season, no significant differences were observed for plant height (PH), length of spikes (LS), spikelet number per spike (SNPS), kernel number per spike (KNPS), 1000 kernel weight (TKW), and kernel weight per spike (KWPS) between the wheat parent CN25 and the T6RS.6AL translocation line 117-6. However, a significant increase (*p* < 0.05) was observed for spike number per plant (SNPP) and kernel weight per plant (KWPP) between them. The SNPP and KWPP of 117-6 were 10.0 ± 1.0 spikes per plant and 33.8 ± 1.2 g per plant, higher than those of CN25 by 85.7% and 72.4%, respectively. In the 2021 to 2022 crop season, no significant differences were observed for PH, LS, TKW, and SNPP between CN25 and 117-6. In addition, a significant increase (*p* < 0.05) was observed for SNPS, KNPS, KWPP, and KWPS. The results indicated that the introduction of the 6RS chromosome of Qinling rye can significantly increase the agronomic traits, as well as increase the resistance to stripe rust and powdery mildew in common wheat.

## 3. Discussion

### 3.1. New 6RS Chromosome Arm with Resistance to Pst and Bgt Originating from Qinling Rye

Related species of wheat play an important role in the genetic improvement of wheat [38]. Many chromosome fragments of different related species have been introduced into the wheat genome through chromosome translocation, substitution, or addition [9,10,11,12,13,14,15,16,17,18]. However, the most important and useful related species of wheat is rye [8]. The 1RS chromosome arm of rye was first introduced into wheat from Petkus rye in the 1950s in Germany via wheat-rye 1RS.1BL translocation [8,39]. A number of useful genes on 1RS, such as *Yr9* (resistance to stripe rust) and *Pm8* (resistance to powdery mildew), were transferred into the wheat genome through this translocation chromosome [21]. Moreover, some yield-enhanced factors were also located on the 1RS chromosome arm [20]. Therefore, 1RS.1BL translocation lines are widely used in wheat breeding programs worldwide [39,40]. Unfortunately, due to the change in the prevalent virulent pathotypes, the *Yr9* and *Pm8* genes were overcome during the 1990s [4]. To solve this problem, several new 1RS.1BL translocations have been developed from different rye sources and have shown resistance to stripe rust and powdery mildew in the past 20 years [4,26,27]. For the more efficient use of rye in wheat breeding, it is also important to introduce chromosomes other than 1RS from rye into the wheat genome. For example, several resistance genes against Hessian fly (*H21*), stem rust (*Sr59*), leaf rust, and powdery mildew on the 2RL chromosome arm of rye were introduced into the wheat genome via the 2RL.2BS, 2RL.2AS, or 2RL.2DS chromosome translocations [41,42,43]. The *Sr27* gene on the rye 3RS chromosome arm was transferred into the wheat genome via 3RS.3AL or 3RS.3BL chromosome translocation [44]. There were 287 disease resistance-associated genes mapped on the 6R chromosome [28]. Breeders paid more attention to the use of the 6R chromosome because there were many excellent genes on the 6R chromosome of rye that can be used for the genetic improvement of wheat [9,31,32,33,34,35,36,37,45,46,47]. The 6R chromosome of rye has also been transferred from different rye varieties into the wheat genome. Many studies have indicated that the 6R chromosome provides resistance to powdery mildew. For example, An et al. [37] reported that the 6R disomic addition line WR-49-1, which originated from the rye cultivar German White, showed resistance to powdery mildew. Du et al. [48] reported a wheat-rye 6RL small segment translocation line with resistance to powdery mildew, which originated from the rye cultivar Kustro. Han et al. [9] also reported a wheat-rye 6R addition line YT2, which showed resistance to powdery mildew. The 6R chromosome may contain stripe rust resistance genes [49]. For example, Li et al. [50] reported that the wheat-rye 6R(6D) substitution line HH41, which originated from the wild *Secale* species *Secale africanum* Stapf, showed resistance to powdery mildew and stripe rust. Schneider et al. [51] reported that 6R disomic addition lines that originated from the rye cultivar Kriszta showed resistance to stripe rust. Many useful resistance genes were also mapped on the rye 6R chromosome. For example, *Yr83* and *Pm20,* were mapped on the 6RL chromosome arm [32,34], and *Pm56* was mapped on 6RS [33]. However, the research on 6R is still dominated by whole chromosome substitution or chromosome addition [50,51], and the research on 6RL is far more extensive than that on 6RS [32,34,45,48]. Moreover, few translocations involving the chromosome 6R of rye and wheat have been reported [33], and it has not been reported that 6RS is resistant to both stripe rust and powdery mildew.

In recent years, there have been several effective methods to transfer alien chromosomes into the wheat genome. The chromosome segments of related species could be transferred into the wheat genome using radiation [46,52], the *ph1b* mutant [33,53], incorporating recessive crossability alleles (*kr1kr1kr2kr2*) [54], or through alien monosomic additions [4,26]. Monosomic addition chromosomes can effectively induce the imbalance of the chromosomes, leading to chromosome mismatch, improper chromosome division, and chromosome loss [55,56,57]. Finally, chromosome translocation, substitution, and small fragment translocation could be formed [4,26,30,58]. In the present study, a 6R monosomic addition line was selected and used as a tool to transfer the 6R chromosome into the wheat genome, and a new T6RS.6AL translocation line, 117-6, was selected (Figure 1). According to the development of other translocation lines in our laboratory, it is indeed possible to select the corresponding translocation lines quickly by using monosomic addition lines.

Qinling rye, which was a land race rye of China, was collected by our lab in the 1990s. The ISSR results indicated that Qinling rye is very different from other cultivated rye, with genetic similarity indices from 0.7215 to 0.8608 [25]. Since China has no tradition of growing rye, there may be more genetic diversity than with the long-domesticated rye of Europe. The genome of Chinese rye may be quite different from that of European rye [28,59,60]. Therefore, Chinese rye may contain more favorable genes that can be used for wheat genetic improvement. In our previous study, several Chinese ryes, such as Weining, Baili, and Aigan, collected from southwest or northwest China, exhibited very high genetic diversity [61], and they may contain a large number of stripe rust resistance genes [26]. Like them, Qinling rye may contain a large number of favorable genes, which will play an important role in the genetic improvement of wheat. There have been several reports of T6RS.6AL translocation [33]. However, the T6RS.6AL translocation line developed in this study showed high resistance to both stripe rust and powdery mildew in both the seedling stage and adult stage (Table 1). It is different from the T6RS.6AL translocation reported previously (only resistance to *Bgt*) [33]. The agronomic traits between them were also different, and 117-6 showed better performance for agronomic traits [62]. The results indicated that the T6RS.6AL translocation line reported in this study is a new translocation line. The cytogenetic analysis in the BC_2_F_6_ generation also indicated that resistance to stripe rust and powdery mildew was conferred by the 6RS chromosome arm, which was from Qinling rye (Figure 6, Table 2).

### 3.2. Breeding Value of the T6RS.6AL Translocation Line 117-6

In recent years, many excellent resistance genes of wheat have gradually lost resistance, and the scarcity of wheat intraspecies genetic resources has become an important bottleneck limiting the breakthrough of wheat genetic improvement [5,26,63,64,65]. Rye is one of the tertiary gene sources of wheat, has many special and excellent genes, and is a huge gene pool for improving common wheat, such as great adaptability to the environment, resistance to diseases and pests, strong tillering ability, large spikes, greater number of spikelets, and higher protein content [28,59,60]. Therefore, rye is currently one of the most successful species used in wheat genetic improvement [39,40,62]. An excellent example of the use of rye in wheat genetic improvement and wheat breeding programs is the T1RS.1BL translocation [23,40]. The T1RS.1BL translocation conferred wheat with a great disease resistance ability and a high yield [23]. Therefore, T1RS.1BL translocations have been used worldwide. However, due to the quick change in the prevalent pathogens *Pst* and *Bgt*, the resistance genes on 1RS were overcome by new pathogens at the end of the last century [4]. Moreover, the 1RS arm in wheat has end-use quality defects that are partially attributable to the presence of ω-secalins, which are encoded by genes at the *Sec-1* locus of the rye 1RS chromosome arm [63]. Therefore, an increasing number of scientists and breeders are focusing on other chromosomes in rye. However, it is difficult to find or develop new germplasms that are resistant to multiple diseases and that have good agronomic traits. In this study, a new wheat-rye T6RS.6AL translocation line, 117-6, was developed, which showed high resistance to the most severe diseases of wheat, namely, stripe rust and powdery mildew (Table 1). Meanwhile, 117-6 exhibited excellent agronomic traits with high yield (Table 3). In the 2020–2021 crop season, the SNPP of 117-6 was significantly higher than the wheat parent CN25. In the 2021–2022 crop season, the SNPP of 117-6 was still higher than that of CN25, but not significant. However, the KNPS, SNPS, KWPS, and KWPP were significantly higher than those of CN25 (Table 3). This effectively makes up for the reduced number of spikes. It was indicated that 117-6 can effectively regulate the relationship between spike number and grain traits. In addition, this is also a very important trait after rye chromatin was introduced into the wheat genome. This new germplasm would be a useful alternate genetic resource for wheat breeding programs in the future.

## 4. Materials and Methods

### 4.1. Plant Materials

A common wheat genotype, Chuannong25 (CN25), was used as the receptor of distant crosses. CN25 was a high-yield wheat cultivar, and was released by the Sichuan Provincial Variety Examination and Committee in 2007. The pure genetic stocks of CN25 used in this study were bred through single-spike descent over several generations. The rye donor Qinling was collected by our laboratory in China in the 1990s. CN25 was crossed directly with Qinling rye to develop new translocation lines according to the methods of Ren et al. [26,30]. The F_1_ seedlings were soaked in 0.05% colchicine plus 3% dimethyl sulfoxide for 8 h to produce the amphidiploid (C_1_). The C_1_ plants were then backcrossed to CN25 twice to produce BC_2_F_0_ seeds. The seeds were then reproduced via selfing. Only the plants that showed resistance to diseases in the field would be harvested and reproduced in the continued generations. In BC_2_F_6_, the root tips of the seedling were examined via ND-FISH before sowing, and a new T6RS.6AL translocation line was identified and selected. The details of the development process of translocation lines were described by Ren et al. [26,30].

### 4.2. Cytogenetic and Molecular Analyses

All plant materials used in this study were identified through both cytogenetic and molecular analyses. ND-FISH was used to distinguish wheat chromosomes, and to detect rye chromosomes in the wheat genome. Five oligonucleotide probes, Oligo-pSc119.2-1 (5′-CCGTTTTGTGGACTATTACTCACCGCTTTGGGGTCCCATAGCTAT-3′), Oligo-pTa535-1 (5′-AAAAACTTGACGCACGTCACGTACAAATTGGACAAACTCTTTCGGAGTATCAGGGTTTC-3′), Oligo-Ku (5′-GATCGAGACTTCTAGCAATAGG CAAAAATAGTAATGGTATCCGGGTTCG-3′), Oligo-pSc200 (5′-CTCACTTGCTTTGAGAGTCTCGATCAATTCGGACTCTAGGTTGATTTTTGTATTTTCT-3′), and Oligo-pSc250 (5′-TGTGTTGTTCTTGGACAAAACAATGCATACCATCTCTTCTAC-3′), were mixed and used together in one ND-FISH experiment [66]. Oligo-pSc119.2-1 can distinguish the B-genome and the 4A, 5A, 2D, 3D, and 4D chromosomes of wheat, as well as the genome of rye [66,67,68]. Oligo-pTa535-1 can distinguish the D-genome and the 1A, 2A, 3A, 4A, 6A, 7A, 3B, 6B, and 7B chromosomes of wheat [66,67,68]. The combination of Oligo-Ku, Oligo-pSc200, and Oligo-pSc250 can distinguish rye chromosomes in the wheat genetic background [66]. Therefore, the combination of these five probes can accurately and effectively identify wheat-rye translocation chromosomes in the wheat genome. The centromeric-specific probe Oligo-CCS1 (5′-CCGTTTGATAGAGGCAAAGGTGTCCCGTCTTTTGATGAGA-3′), rye centromeric-specific probe Oligo-PAWRC.1 (5′-CCGTTTGATAGAGGCAAAGGTGTCCCGTCTTTTGATGAGA-3′), and telomere-specific probe Oligo-Telo (5′-TTTAGGGTTTAGGGTTTAGGG-3′) were mixed and used in another ND-FISH experiment to detect the composition of the centromere and telomere [67,68]. Moreover, the translocation line was also identified using GISH to determine the alien chromosomes. The genomic DNA of Qinling rye was used as the probe, and GISH was performed according to the methods of Ren et al. [58]. Cell images were captured using an epifluorescence microscope (model BX51, Olympus, Center Valley, PA, USA) equipped with a cooled charge-coupled device camera and operated with the software program HCImage Live (version 2.0.1.5, Hamamatsu Corp., Sewickley, PA, USA). Probe labeling and ND-FISH were performed as previously described [48,66].

All plant materials used in this study were also identified using specific molecular markers. The genomic DNA of the plant materials was isolated from young leaves using the surfactant cetyltrimethylammonium bromide (CTAB) [69]. Two specific molecular markers were used in this study. The primer pairs KU88 (F: 5′-CAGGATATCCCACAACACAAGA-3′; R: 5′-ATGGGTTGTATTTGCCGAAA-3′) and KU291 (F: 5′-GAGACTACCCGTCGAAGGAC-3′; R: 5′-GGGGCTTCATCGACAATCTA-3′) were used to detect 6RS in rye, and can amplify a specific approximately 400 bp fragment band from the rye 6RS chromosome arm [70]. The PCR was performed according to Qiu et al. [70].

### 4.3. Resistance Analysis

All plant materials used in this study were examined for resistance to stripe rust and powdery mildew, which are devastating diseases of wheat in China in greenhouses and fields. CYR32, CYR33, and CYR34 are considered the most virulent and frequent *Pst* races occurring in Chinese wheat planted areas [26,65]. These three *Pst* races were used in stripe rust resistance tests in the seedling stage. CYR32 is virulent toward *Yr1, 2, 3, 4, 6, 7, 8, 9, 11, 12, 13, 14, 16, 17, 18, 25, 27, 28, 29, 30, 31, 32, 43, 44, A, Alba, Cle, CV, Gaby, Res, SD, SO, Exp2, Sk*, and *SpP*. CYR33 is virulent toward *Yr1, 2, 3, 4, 6, 7, 8, 9, 11, 12, 17, 18, 25, 28, 29, 30, 31, 32, A*, and *Su*. CYR34 is virulent toward *Yr1, 2, 3, 4, 6, 7, 8, 9, 10, 11, 12, 13, 14, 16, 17, 18, 19, 24, 25, 26, 27, 28, 29, 30, 31, 32, 43, 44, Exp2, Sp, A*, and *Sk* [26]. All *Pst* races were provided by the Plant Protection Institute, Gansu Academy of Agricultural Sciences, China. Race E20 of *Bgt* was provided by the Department of Plant Protection, Sichuan Agricultural University, and many newly released wheat cultivars were susceptible to E20 [71]. The inoculation of *Pst* and *Bgt* on wheat seedlings was performed according to the methods described by Ren et al. [30] and Yang et al. [71], with three replications in the greenhouse.

The resistance of all plant materials to *Pst* and *Bgt* was also tested in the field without artificial inoculation at the Qionglai experimental farm station of Sichuan Agricultural University in Chengdu Basin, China (30°25′ N, 103°28′ E) from 2020 to 2022, where the climate is temperate and rainy with a serious prevalence of stripe rust and powdery mildew. Entries were arranged in a randomized complete block design with three replicates. The plot consisted of two rows with a length of 1 m, a row spacing of 25 cm, and a plant spacing of 0.1 m. All plant materials were naturally infected by *Pst* and *Bgt* during the heading to flowering period, and disease reactions were scored at the heading stage for powdery mildew and at the grain filling stage for stripe rust [72,73].

For stripe rust, infection types (ITs) were scored based on a 0–9 scale: ITs 0–3 are resistant, ITs 4–6 are intermediate resistant, and ITs 7–9 are susceptible [26,73]. For powdery mildew, ITs were scored based on a 0–4 scale according to Ren et al. [30] and Xie et al. [74]. Wheat parent CN25 and rye parent Qinling were used as controls.

### 4.4. Agronomic Trait Analysis

All plants were grown at the Qionglai experimental farm station in the crop seasons from 2020 to 2022. The plants were sown at a density of 160 seedling/m^2^ in one-meter-long plots with four rows spaced 25 cm apart, and three replications with randomized complete blocks were used. The plant height (PH), length of spikes (LS), spikelet number per spike (SNPS), spike number per plant (SNPP), kernel weight per spike (KWPS), kernel weight per plant (KWPP), 1000 kernel weight (TKW), and kernel number per spike (KNPS) were determined according to the methods of Ren et al. [62] and Kim et al. [75]. The farm experiments followed the standard wheat cultivation procedures on the Chengdu Plain, and fungicide was used to control diseases and pests [62].

Analysis of variance was performed on the data for each characteristic. The least significant differences (LSD) test was used for means comparisons using Sigmaplot 2001 software (SPSS Inc., Chicago, IL, USA).

## Figures and Tables

**Table 1:** Analysis of resistance to stripe rust and powdery mildew when inoculated with epidemic races of *Pst* and *Bgt*.

Lines	Chromosome Type	*Pst* Analysis							*Bgt* Analysis		
		CYR32	CYR33	CYR34	In Field			E20	In Field		
					2020	2021	2022		2020	2021	2022
CN25	6A	4	3	5	5	7	5	4	4	4	4
117-6	T6RS.6AL	0	0	0	0	0	0	0	0	0	0
Qinling rye	6R	0	0	0	0	0	0	0	0	0	0

Infection type for stripe rust: 0: no visible symptoms; 1: necrotic flecks; 2: necrotic areas without sporulation; 3: necrotic and chlorotic areas with restricted sporulation; 4–6: moderate uredinia with necrosis and chlorosis; 7–8: abundant uredinia with chlorosis; 9: abundant uredinia without chlorosis. Resistant: IT 0–3; intermediate resistant: IT 4–6; HS: highly susceptible: IT 7–9. Infection type for powdery mildew: 0: no visible symptoms; 0;: hypersensitive necrotic flecks; 1: minute colonies with few conidia produced; 2: colonies with moderately developed hyphae but few conidia; 3: colonies with well-developed hyphae and abundant conidia but colonies not joined together; 4: colonies with well-developed hyphae and abundant conidia, and colonies mostly joined together.

**Table 2:** Relationship between resistance ability and chromosome types in BC_2_F_6_ in the field in 2020.

Chromosome Types	Resistance to *Pst*	Resistance to *Bgt*
T6RS.6AL (117-6)	0	0
6R monosomic addition plants	0	0
6RS addition plants	0	0
6R disomic addition plants	0	0
Heterozygous T6RS.6AL plants	0	0
Plants that lost 6R (AABBDD)	5	4
CN25 (AABBDD, control)	5	4

**Table 3:** Comparisons of agronomic traits between 117-6 and its wheat parent CN25.

Lines	PH	LS	SNPS	KNPS	KWPP	TKW	SNPP	KWPS
CN25(2021)	93.8 ± 2.7	13.4 ± 0.9	22.4 ± 1.1	55.3 ± 5.9	18.2 ± 4.7	56.0 ± 2.0	5.8 ± 0.8	3.1 ± 0.4
117-6(2021)	93.7 ± 2.5	12.8 ± 0.8	21.3 ± 0.6	59.7 ± 5.4	33.8 ± 1.2 *	56.8 ± 1.3	10.0 ± 1.0 *	3.4 ± 0.3
CN25(2022)	88.0 ± 2.0	12.5 ± 0.5	21.4 ± 0.5	57.1 ± 2.5	14.2 ± 4.7	51.5 ± 1.3	4.8 ± 0.7	2.9 ± 0.1
117-6(2022)	85.6 ± 4.3	13.3 ± 0.1	22.6 ± 0.5 *	72.4 ± 7.7 *	22.4 ± 4.7 *	53.7 ± 5.4	5.8 ± 1.5	3.9 ± 0.5 *

PH: plant height (cm); LS: length of spikes (cm); SNPS: spikelet number per spike; KNPS: kernel number per spike; KWPP: kernel weight per plant; TKW: 1000 kernel weight (g); SNPP: spike number per plant; KWPS: kernel weight per spike. *: Significant difference at *p* = 0.05.

## Data Availability

Not applicable.

## References

[1] Chaves M.S., Martinelli J.A., Wesp-Guterres C., Graichen F.A., Brammer S.P., Scagliusi S.M., da Silva P.B., Wiethölter P., Torres G.A.M., Lau E.Y. (2013). The importance for food security of maintaining rust resistance in wheat. Food Secur..

[2] Ren T., Fan T., Chen S., Ou X., Chen Y., Jiang Q., Diao Y., Sun Z., Peng W., Ren Z. (2021). QTL Mapping and validation for kernel area and circumference in common wheat via high-density SNP-based genotyping. Front. Plant Sci..

[3] Chen W.Q., Wellings C., Chen X.M., Kang Z.S., Liu T.G. (2014). Wheat stripe (yellow) rust caused by *Puccinia striiformis* f. sp. *tritici*. Mol. Plant Pathol..

[4] Ren T.H., Yang Z.J., Yan B.J., Zhang H.Q., Fu S.L., Ren Z.L. (2009). Development and characterization of a new 1BL.1RS translocation line with resistance to stripe rust and powdery mildew of wheat. Euphytica.

[5] Ren T., Li Z., Tan F., Jiang C., Luo P., Oliver R. (2021). Advances in identifying stripe rust resistance genes in cereals. Achieving Durable Disease Resistance in Cereals.

[6] Wan A., Chen X., He Z. (2007). Wheat stripe rust in China. Aust. J. Agric. Res..

[7] Hou Z.G., Liu W.C., Shao Z.R., Jiang R.Z. (2000). On developing long-term meteorological prediction research of crops pests and diseases prevailing in China. J. Nat. Disasters.

[8] Schlegel R., Korzun V. (1997). About the origin of 1RS.1BL wheat–rye chromosome translocations from Germany. Plant Breed..

[9] Han G., Yan H., Wang J., Cao L., Liu S., Li X., Zhou Y., Fan J., Li L., An D. (2022). Molecular cytogenetic identification of a new wheat-rye 6R addition line and physical localization of its powdery mildew resistance gene. Front. Plant Sci..

[10] Li Z., Ren Z., Tan F., Tang Z., Fu S., Yan B., Ren T. (2016). Molecular cytogenetic characterization of new wheat-rye 1R(1B) substitution and translocation lines from a Chinese *Secale cereal* L. *Aigan* with resistance to stripe rust. PLoS ONE.

[11] Chen P.D., Qi L.L., Zhou B., Zhang S.Z., Liu D.J. (1995). Development and molecular cytogenetic analysis of wheat-*Haynaldia villosa* 6VS/6AL translocation lines specifying resistance to powdery mildew. Theor. Appl. Genet..

[12] Luo P.G., Luo H.Y., Chang Z.J., Zhang H.Y., Zhang M., Ren Z.L. (2009). Characterization and chromosomal location of *Pm40*, in common wheat: A new gene for resistance to powdery mildew derived from *Elytrigia intermedium*. Theor. Appl. Genet..

[13] Wang X., Han B., Sun Y., Kang X., Zhang M., Han H., Zhou S., Liu W., Lu Y., Yang X. (2022). Introgression of chromosome 1P from *Agropyron cristatum* reduces leaf size and plant height to improve the plant architecture of common wheat. Theor. Appl. Genet..

[14] Ma P., Xu H., Xu Y., Li L., Qie Y., Luo Q., Zhang X., Li X., Zhou Y., An D. (2015). Molecular mapping of a new powdery mildew resistance gene *Pm2b* in Chinese breeding lineKM2939. Theor. Appl. Genet..

[15] Cao Z., Deng Z., Wang M., Wang X., Jing J., Zhang X., Shang H., Li Z. (2008). Inheritance and molecular mapping of an alien stripe-rust resistance gene from a wheat-*Psathyrostachys huashanica* translocation line. Plant Sci..

[16] Marais G.F., Mccallum B., Snyman J.E., Pretorius Z.A., Marais A.S. (2005). Leaf rust and stripe rust resistance genes *Lr54* and *Yr37* transferred to wheat from *Aegilops kotschyi*. Plant Breed..

[17] Liu J., Chang Z., Zhang X., Yang Z., Li X., Jia J. (2013). Putative *Thinopyrum intermedium*-derived stripe rust resistance gene *Yr50* maps on wheat chromosome arm 4BL. Theo. Appl. Genet..

[18] Bao Y., Wang J., He F., Ma H., Wang H. (2012). Molecular cytogenetic, identification of a wheat (*Triticum aestivum*)-American dune grass (*Leymus mollis*) translocation line resistant to stripe rust. Genet. Mol. Res..

[19] Sharma P., Chaudhary H.K., Kapoor C., Manoj N.V., Singh K., Sood V.K.A. (2021). Molecular cytogenetic analysis of novel wheat-rye translocation lines and their characterization for drought tolerance and yellow rust resistance. Cereal Res. Comm..

[20] Howell T., Hale I., Jankuloski L., Bonafede M., Gilbert M., Dubcovsky J. (2014). Mapping a region within the 1RS.1BL translocation in common wheat affecting grain yield and canopy water status. Theor. Appl. Genet..

[21] Mago R., Miah H., Lawrence G.J., Wellings C.R., Spielmeyer W., Bariana H.S., McIntosh R.A., Pryor A.J., Ellis J.G. (2005). High-resolution mapping and mutation analysis separate the rust resistance genes *Sr31*, *Lr26* and *Yr9* on the short arm of rye chromosome 1. Theor. Appl. Genet..

[22] Ren T., Tang Z., Fu S., Yan B., Tan F., Ren Z., Li Z. (2017). Molecular cytogenetic characterization of novel wheat-rye T1RS.1BL translocation lines with high resistance to diseases and great agronomic traits. Front. Plant Sci..

[23] Rabinovich S.V. (1998). Importance of wheat–rye translocations for breeding modern cultivars of *Triticum aestivum* L.. Euphytica.

[24] Gong W., Han R., Ren T., Wang C., Yang Z., Yan M., Luo P., Liu A., Li H., Liu C. (2020). Molecular detection of 1RS/1BL translocation and stripe rust resistance gene *Yr41* in 1293 wheat cultivars (lines). Shandong Agric. Sci..

[25] Ren T.H., Chen F., Zou Y.T., Jia Y.H., Zhang H.Q., Yan B.J., Ren Z.L. (2011). Evolutionary trends of microsatellites during the speciation process and phylogenetic relationships within the genus *Secale*. Genome.

[26] Ren T., Jiang Q., Sun Z., Zhao L., Peng W., Ren Z., Tan F., Luo P., Li Z. (2022). Development and molecular cytogenetic characterization of novel primary wheat-rye 1RS.1BL translocation lines from multiple rye sources with resistance to stripe rust. Plant Dis..

[27] Han G.H., Liu S.Y., Wang J., Jin Y.L., Zhou Y.L., Luo Q.L., Liu H., Zhao H., An D.G. (2020). Identification of an elite wheat-rye T1RS∙1BL translocation line conferring high resistance to powdery mildew and stripe rust. Plant Dis..

[28] Li G., Wang L., Yang J., He H., Jin H., Li X., Ren T., Ren Z., Li F., Han X. (2021). A high-quality genome assembly highlights rye genomic characteristics and agronomically important genes. Nat. Genet..

[29] Liu C., Wang J., Fu S., Wang L., Li H., Wang M., Huang Y., Shi Q., Zhou Y., Guo X. (2022). Establishment of a set of wheat-rye addition lines with resistance to stem rust. Theor. Appl. Genet..

[30] Ren T., Sun Z., Ren Z., Tan F., Luo P., Tang Z., Fu S., Li Z. (2020). Molecular and cytogenetic characterization of a wheat-rye 7BS.7RL translocation line with resistance to stripe rust, powdery mildew and Fusarium head blight. Phytopathology.

[31] Friebe B., Kynast R.G., Hatchett J.H., Sears R.G., Wilson D.L., Gill B.S. (1999). Transfer of wheat-rye translocation chromosomes conferring resistance to Hessian Fly from bread wheat into durum wheat. Crop Sci..

[32] Friebe B., Heun M., Tuleen N., Zeller F.J., Gill B.S. (1994). Cytogenetically monitored transfer of powdery mildew resistance from rye into wheat. Crop Sci..

[33] Hao M., Liu M., Luo J., Fan C., Yi Y., Zhang L., Yuan Z., Ning S., Zheng Y., Liu D. (2018). Introgression of powdery mildew resistance gene *Pm56* on rye chromosome arm 6RS into wheat. Front. Plant Sci..

[34] Li J., Dundas I., Dong C., Li G., Trethowan R., Yang Z., Hoxha S., Zhang P. (2020). Identification and characterization of a new stripe rust resistance gene *Yr83* on rye chromosome 6R in wheat. Theor. Appl. Genet..

[35] Duan Y., Luo J., Yang Z., Li G., Tang Z., Fu S. (2022). The physical location of stripe rust resistance genes on chromosome 6 of Rye (*Secale cereale* L.) AR106BONE. Front. Plant Sci..

[36] Grdzielewska A., Milczarski P., Molik K., Pawowska E. (2020). Identification and mapping of a new recessive dwarfing gene *dw9* on the 6RL rye chromosome and its phenotypic effects. PLoS ONE.

[37] An D., Zheng Q., Luo Q., Ma P., Zhang H., Li L., Han F., Xu H., Xu Y., Zhang X. (2015). Molecular cytogenetic identification of a new wheat-rye 6R chromosome disomic addition line with powdery mildew resistance. PLoS ONE.

[38] Friebe B., Jiang J., Raupp W.J., McIntosh R.A., Gill B.S. (1996). Characterization of wheat-alien translocations conferring resistance to diseases and pests: Current status. Euphytica.

[39] Baum M., Appels R. (1991). The cytogenetic and molecular architecture of chromosome 1R-one of the most widely utilized sources of alien chromatin in wheat varieties. Chromosoma.

[40] Lelley T., Eder C., Grausgruber H. (2004). Influence of 1BL.1RS wheat–rye chromosome translocation on genotype by environment interaction. J. Cereal Sci..

[41] Friebe B., Hatchett J.H., Sears R.G., Gill B.S. (1990). Transfer of Hessian fly resistance from ‘Chaupon’ rye to hexaploid wheat via a 2BS/2RL wheat-rye chromosome translocation. Theor. Appl. Genet..

[42] Hysing S.C., Hsam S.L.K., Singh R.P., Huerta-Espino J., Boyd L.A., Koebner R.M.D., Cambron S., Johnson J.W., Bland D.E., Liljeroth E. (2007). Agronomic performance and multiple disease resistance in T2BS.2RL wheat-rye translocation lines. Crop Sci..

[43] Rahmatov M., Rouse M.N., Nirmala J., Danilova T., Friebe B., Steffenson B.J., Johansson E. (2016). A new 2DS·2RL Robertsonian translocation transfers stem rust resistance gene Sr59 into wheat. Theor. Appl. Genet..

[44] Marais G.F., Marais A.S. (1994). The derivation of compensating translocations involving homoeologous group 3 chromosomes of wheat and rye. Euphytica.

[45] Li M., Tang Z., Qiu L., Wang Y., Tang S., Fu S. (2016). Identification and physical mapping of new PCR-based markers specific for the long arm of rye (*Secale cereale* L.) Chromosome 6. J. Genet. Genom..

[46] Mukai Y., Friebe B., Hatchett J.H., Yamamoto M., Gill B.S. (1993). Molecular cytogenetic analysis of radiation-induced wheat-rye terminal and intercalary chromosomal translocations and the detection of rye chromatin specifying resistance to Hessian fly. Chromosoma.

[47] Dundas I.S., Frappell D.E., Crack D.M., Fisher J.M. (2001). Deletion mapping of a nematode resistance gene on rye chromosome 6R in wheat. Crop Sci..

[48] Du H., Tang Z., Duan Q., Tang S., Fu S. (2018). Using the 6RL^Ku^ minichromosome of rye (*Secale cereale* L.) to create wheat-rye 6D/6RL^Ku^ small segment translocation lines with powdery mildew resistance. Int. J. Mol. Sci..

[49] Miller T.E. (1984). The homoeologous relationships between the chromosome of rye and wheat. Current status. Can. J. Genet. Cytol..

[50] Li G., Tang L., Yin Y., Zhang A., Yu Z., Yang E., Tang Z., Fu S., Yang Z. (2020). Molecular dissection of *Secale africanum* chromosome 6Rafr in wheat enabled localization of genes for resistance to powdery mildew and stripe rust. BMC Plant Biol..

[51] Schneider A., Rakszegi M., Molnár-Láng M., Szakács É. (2016). Production and cytomolecular identification of new wheat-perennial rye (*Secale cereanum*) disomic addition lines with yellow rust resistance (6R) and increased arabinoxylan and protein content (1R, 4R, 6R). Theor. Appl. Genet..

[52] Chen P., You C., Hu Y., Chen S., Zhou B., Cao A., Wang X. (2013). Radiation-induced translocations with reduced *Haynaldia villosa* chromatin at the *Pm21* locus for powdery mildew resistance in wheat. Mol. Breed..

[53] Sears E.R. (1977). An induced mutant with homoeologous pairing in common wheat. Can. J. Genet. Cytol..

[54] Molnár-Láng M., Cseh A., Szakács E., Molnár I. (2010). Development of a wheat genotype combining the recessive crossability alleles *kr1kr1kr2kr2* and the 1BL.1RS translocation, for the rapid enrichment of 1RS with new allelic variation. Theor. Appl. Genet..

[55] Tan G.X. (2008). Monosomic alien addition lines: A new tool for studying and using plant genomics. Yi Chuan.

[56] Jena K.K., Khush G.S. (1989). Monosomic alien addition lines of rice: Production, morphology, cytology, and breeding behavior. Genome.

[57] Fu S., Sun C., Yang M., Fei Y., Tan F., Yan B., Ren Z., Tang Z. (2013). Genetic and epigenetic variations induced by wheat-rye 2R and 5R monosomic addition lines. PLoS ONE.

[58] Ren T., Li Z., Yan B., Tan F., Tang Z., Fu S., Yang M., Ren Z. (2017). Targeted segment transfer from rye chromosome 2R to wheat chromosomes 2A, 2B, and 7B. Cytogenet. Genome Res..

[59] Rabanus-Wallace M.T., Hackauf B., Mascher M., Lux T., Wicher T., Gundlash H., Baez M., Houben A., Mayer K.F.X., Guo L. (2021). Chromosome-scale genome assembly provides insights into rye biology, evolution and agronomic potential. Nat. Genet..

[60] Sun Y., Shen E., Hu Y., Wu D., Feng Y., Lao S., Dong C., Du T., Hua W., Ye C. (2022). Population genomic analysis reveals domestication of cultivated rye from weedy rye. Mol. Plant.

[61] Ren T.H., Chen F., Yan B.J., Zhang H.Q., Ren Z.L. (2012). Genetic diversity of wheat-rye 1BL.1RS translocation lines derived from different wheat and rye sources. Euphytica.

[62] Li Q., Huang L., Li Y., Fan C., Xie D., Zhao L., Zhang S., Chen X., Ning S., Yuan Z. (2020). Genetic stability of wheat rye 6RS/6AL translocation chromosome and its transmission through gametes. Acta Agric. Sin..

[63] Lukaszewski A.J. (2006). Cytogenetically engineered rye chromosomes 1R to improve bread-making quality of hexaploid triticale. Crop Sci..

[64] Müller M.C., Kunz L., Schudel S., Lawson A.W., Kammerecker S., Isaksson J., Wyler M., Graf J., Sotiropoulos A.G., Praz C.R. (2022). Ancient variation of the *AvrPm17* gene in powdery mildew limits the effectiveness of the introgressed rye *Pm17* resistance gene in wheat. Proc. Natl. Acad. Sci. USA.

[65] Wang N., Tang C., Fan X., He M., Gan P., Zhang S., Hu Z., Wang X., Yan T., Shu W. (2022). Inactivation of a wheat protein kinase gene confers broad-spectrum resistance to rust fungi. Cell.

[66] Ren T., He M., Sun Z., Tan F., Luo P., Tang Z., Fu S., Yan B., Ren Z., Li Z. (2019). The Polymorphisms of oligonucleotide probes in wheat cultivars determined by ND-FISH. Molecules.

[67] Cuadrado Á., Jouve N. (2010). Chromosomal detection of simple sequence repeats (SSRs) using nondenaturing FISH (ND-FISH). Chromosoma.

[68] Tang Z., Yang Z., Fu S. (2014). Oligonucleotides replacing the roles of repetitive sequences pAs1, pSc119.2, pTa-535, pTa71, CCS1, and pAWRC.1 for FISH analysis. J. Appl. Genet..

[69] Doyle J.J., Doyle J.L. (1987). A rapid DNA isolation procedure from small quantities of fresh leaf tissues. Phytochem. Bull..

[70] Qiu L., Tang Z., Li M., Fu S. (2016). Development of new PCR-based markers specific for chromosome arms of rye (*Secale cereal* L.). Genome.

[71] Yang H., Zhong S., Chen C., Yang H., Chen W., Tan F., Zhang M., Chen W., Ren T., Li Z. (2021). Identification and cloning of a CC-NBS-NBS-LRR gene as a candidate of *Pm40* by integrated analysis of both the available transcriptional data and published linkage mapping. Int. J. Mol. Sci..

[72] Ren T., Ren Z., Yang M., Yan B., Tan F., Fu S., Tang Z., Li Z. (2018). Novel source of 1RS from Baili rye conferred high resistance to diseases and enhanced yield traits to common wheat. Mol. Breed..

[73] Wan A., Zhao Z., Chen X., He Z., Jin S., Jia Q., Yao G., Yang J., Wang B., Li G. (2004). Wheat stripe rust epidemic and virulence of *Puccinia striiformis* f. sp. *tritici* in China in 2002. Plant Dis..

[74] Xie C.J., Sun Q.X., Ni T., Nevo E., Fahima F. (2004). Identification of resistance gene analogue markers closely linked to wheat powdery mildew resistance gene *Pm31*. Plant Breed..

[75] Kim W., Johnson J.W., Baenziger P.S., Lukaszewski A.J., Gaines C.S. (2004). Agronomic effect of wheat–rye translocation carrying rye chromatin (1R) from different sources. Crop Sci..

